# Cell Death and Serum Markers of Collagen Metabolism during Cardiac Remodeling in *Cavia porcellus* Experimentally Infected with *Trypanosoma cruzi*

**DOI:** 10.1371/journal.pntd.0001996

**Published:** 2013-02-07

**Authors:** Yagahira E. Castro-Sesquen, Robert H. Gilman, Henry Paico, Verónica Yauri, Noelia Angulo, Fredy Ccopa, Caryn Bern

**Affiliations:** 1 Laboratorio de Investigación en Enfermedades Infecciosas, Universidad Peruana Cayetano Heredia, Lima, Peru; 2 Asociación Benéfica PRISMA, Lima, Peru; 3 Department of International Health, Johns Hopkins University, Bloomberg School of Hygiene and Public Health, Baltimore, Maryland, United States of America; 4 School of Veterinary Medicine, Universidad Nacional Mayor de San Marcos, Lima, Peru; 5 Global Health Sciences and Department of Epidemiology and Biostatistics, University of California San Francisco, San Francisco, California, United States of America; US Food and Drug Administration, United States of America

## Abstract

We studied cell death by apoptosis and necrosis in cardiac remodeling produced by *Trypanosoma cruzi* infection. In addition, we evaluated collagen I, III, IV (CI, CIII and CIV) deposition in cardiac tissue, and their relationship with serum levels of procollagen type I carboxy-terminal propeptide (PICP) and procollagen type III amino-terminal propeptide (PIIINP). Eight infected and two uninfected guinea pigs were necropsied at seven time points up to one year post-infection. Cell death by necrosis and apoptosis was determined by histopathological observation and terminal deoxynucleotidyl transferase dUTP nick end labeling, respectively. Deposition of cardiac collagen types was determined by immunohistochemistry and serum levels of PICP, PIIINP, and anti-*T. cruzi* IgG1 and IgG2 by ELISA. IgG2 (Th1 response) predominated throughout the course of infection; IgG1 (Th2 response) was detected during the chronic phase. Cardiac cell death by necrosis predominated over apoptosis during the acute phase; during the chronic phase, both apoptosis and necrosis were observed in cardiac cells. Apoptosis was also observed in lymphocytes, endothelial cells and epicardial adipose tissue, especially in the chronic phase. Cardiac levels of CI, CIII, CIV increased progressively, but the highest levels were seen in the chronic phase and were primarily due to increase in CIII and CIV. High serum levels of PICP and PIIINP were observed throughout the infection, and increased levels of both biomarkers were associated with cardiac fibrosis (p = 0.002 and p = 0.038, respectively). These results confirm the role of apoptosis in cell loss mainly during the chronic phase and the utility of PICP and PIIINP as biomarkers of fibrosis in cardiac remodeling during *T. cruzi* infection.

## Author Summary

Chronic Chagas heart disease (CHHD) caused by the infection with the parasite *Trypanosoma cruzi* is the most important infectious heart disease in the world. The typical manifestations are dilated cardiomyopathy and congestive heart failure; they result from death of cardiomyocytes and their replacement by collagen. Knowing the mechanisms of cardiomyocyte death is important for the development of therapies that prevent them. The contribution of apoptosis in cardiomyocyte death was evaluated in the guinea pig model of *T. cruzi* infection, and the detection of serum levels of collagen precursors were evaluated as biomarkers of cardiac fibrosis. We observed apoptosis of lymphocytes, cardiomyocytes, endothelial cells and epicardial adipose tissue in cardiac tissue of infected guinea pigs. The increase of serum levels of collagen precursors PICP and PIIINP were associated with cardiac fibrosis. Areas of inflammation and apoptosis of epicardial adipose tissue were associated with cardiac pathology, which suggests the importance of epicardial adipose tissue in CCHD. These results show that apoptosis is an important characteristic of cardiac cell death during CCHD and serum levels of PICP and PIIINP could be used as biomarkers of cardiac fibrosis.

## Introduction

Chagas disease, a parasitic infection caused by *Trypanosoma cruzi*, remains a major public health problem in Central and South America with 8 to 10 million people infected [1]. Chronic Chagas heart disease (CCHD), the major clinical consequence, is the most important infectious heart disease in the world, with an estimated 50,000 attributable deaths per year [2].

Many factors have been implicated in the pathogenesis of CCHD, including parasite persistence, cardiac denervation, inflammation, autoimmunity, and microcirculatory changes [3]–[8]. The acute phase of Chagas disease is characterized by the presence of focal necrosis, severe inflammation and abundant amastigote nests in the heart [5]. In the early chronic phase, most infected individuals are asymptomatic, but over a period of years to decades, 30% of patients develop CCHD [3]. The earliest signs of CCHD are usually conduction system abnormalities such as right bundle branch block, with or without multifocal ventricular extrasystoles. Over time, higher-grade conduction deficits and ventricular arrhythmias may occur. The late stage of the disease typically features dilated cardiomyopathy and congestive heart failure. Sudden death may result from arrhythmias, heart block or emboli; patients with advanced cardiomyopathy may die of intractable heart failure [4]. These clinical manifestations result from sequential changes that occur at the cellular level, including lengthening of cardiomyocytes, rearrangement within the myocardial matrix, and death of cardiomyocytes and their replacement by connective tissue; all these changes are part of so-called cardiac remodeling [9].

Different types of cardiomyocyte death have been described, including necrosis, apoptosis, and most recently autophagy, oncosis and programmed necrosis [10]. Necrosis is a well-recognized mechanism of myocardial cell loss during CCHD [3]–[5]. Apoptosis tends to be a chronic process with subtle but harmful impact in different types of cardiac disease [10]. The contribution of apoptosis to cardiomyocyte death in CCHD remains uncertain. A study of chronic chagasic patients with heart failure demonstrated apoptosis in cardiomyocytes and inflammatory cells [11]. However, in another study of patients with chronic chagasic myocarditis, apoptosis was observed in inflammatory cells but not in cardiomyocytes [12]. Apoptosis of endothelial cells, cardiomyocytes and inflammatory cells was observed in the canine model of acute chagasic myocarditis, but dogs were not examined in the chronic phase [13]. In the cardiac tissue of the mouse model, as well as in humans with CCHD, up-regulation of apoptosis related genes such as caspase 12 [14],[15], apoptosis-related Fas antigen, IRF1 and IRF2 [16] has been reported; however, in these studies, the type of cells implicated were not identified [14]–[16].

The prevention of cell death is important to maintain the number of cardiomyocytes and cardiac function. Therapies that regenerate cardiomyocytes using stem cells or progenitor cells show promising results, but clinical trial data are still lacking [17]. Determining the role of apoptosis in myocardial cell loss in CCHD is critical to predicting the impact that interventions at the cellular level might provide in the treatment of heart disease [18].

During cardiac remodeling, cardiomyocyte death leads to replacement by connective tissue, mainly collagen. Collagen determines cardiac structure, function and mechanical properties. Cardiac remodeling during CCHD is characterized by altered collagen turnover and subsequent fibrosis [3], mainly due to an increase of collagen III (CIII) and collagen IV (CIV) [19]. The increase of collagen in cardiac tissue during CCHD is demonstrable in biopsies [20], but noninvasive methods to detect collagen turnover would be useful for prognosis, and in evaluation of therapies and their effect on cardiac remodeling [21]. Many potential biomarkers of fibrosis detect collagen synthesis, such as propeptides of collagen types I and III, procollagen type III amino-terminal propeptide (PIIINP), procollagen type I amino-terminal propeptide (PINP), and procollagen type I carboxy-terminal propeptide (PICP) [21]. Serum levels of PIIINP [21]–[23] and PICP [24] are increased in patients with dilated cardiomyopathy; however, the utility of these biomarkers in CCHD has not previously been evaluated.

We have demonstrated that the guinea pig infection model provides an accurate reflection of the cardiac pathology of Chagas disease in humans [25]. In the current study, we evaluated the role of apoptosis in cell death in the cardiac tissue of guinea pigs during the acute and chronic phases of *T. cruzi* infection. In addition, we evaluated collagen I, III, IV (CI, CIII and CIV) deposition and fibrosis in cardiac tissue, and their relationship with serum levels of PIIINP and PICP during the course of infection.

## Methods

### Ethics statement

The protocol was approved by the San Marcos University Animal Use and Welfare Committee. All experiments adhered to the Guidelines for Animal Experimentation of the Universidad Nacional Mayor de San Marcos.

### Parasites

Trypomastigotes of *T. cruzi* Y strain were donated by Dr. E. Umezawa, Instituto de Medicina Tropical, Universidade de São Paulo, São Paulo, Brazil. The strain was maintained in an *in vitro* culture using LLC-MK_2_ cells following published procedures [26].

### Animals

We used 70 female Andean guinea pigs, weighing 600–700 g (two months old). The animals were sourced from the Pachacamac region of Lima, an area without vector-borne transmission of *T. cruzi*. Prior to parasite inoculation, blood samples were taken from each animal and tested for the presence of anti-*T. cruzi* antibodies and *T. cruzi* DNA; all the animals were negative for both tests. The animals were fed with special food for guinea pigs (cuyina, Purina), alfalfa and water *ad libitum*.

### Experimental infections

Fifty six guinea pigs (experimental group, EG) were inoculated intradermally with 10000 parasites in 100 µl RPMI 1640 medium and 14 guinea pigs (control group, CG) were inoculated with medium alone.

### Sample collection and sacrifice of animals

At 20, 25, 40, 55, 115, 165 and 365 days post inoculation (dpi), eight guinea pigs from EG and two from CG were selected at random, sacrificed by intraperitoneal inoculation of sodium pentobarbital (20 mg/kg) and necropsied. The time points for necropsy were chosen based on previous observations of parasitemia and specific IgM levels in serum [21]. Each time point corresponds to an infection phase as follows: acute phase (20–25 dpi), late acute phase (40–55 dpi), early chronic phase (115–165 dpi) and late chronic phase (365 dpi). Blood samples were collected before sacrifice by cardiac puncture. The cardiac tissue was removed and fixed in absolute ethanol and 10% formalin in PBS. Blood and serum samples were stored at −20°C until use.

### ELISA test for detection of IgG subclasses to *T. cruzi*

Levels of IgG1 and IgG2 against *T. cruzi* were measured by an in-house EAE-ELISA, as described previously [27]. Briefly, ELISA 96 well plates (Immulon 2, Thermo Lab systems, MA) were coated with 3.5 µg/ml (for IgG1 detection) or 2.5 µg/ml (for IgG2 detection) of epimastigote alkaline extract (EAE) antigen and incubated with guinea pig serum 1∶200 dilution. HRP-conjugate was used at a dilution of 1∶ 5000 of goat anti-guinea pig IgG1 or anti-guinea pig IgG2 (ABD Serotec, USA). The plates were incubated with 0.1 mg/well o-phenylene diamine dihydrochloride (Sigma-Aldrich, USA) and 0.05% H_2_O_2_. The OD was determined at 492 nm using a Versa Maxmicroplate spectrophotometer (Molecular Devices Corporation, USA). Each serum sample was analyzed in duplicate.

### DNA extraction and polymerase chain reaction

DNA was purified by proteinase K digestion (Invitrogen, Carlsbad, CA) and phenol-chloroform extraction as previously described [25]. A PCR targeting the kinetoplast DNA of *T. cruzi* was performed using primers 121/122 yielding 330 bp products [28],[29]. Internal control primers specific to guinea pigs (SINEs) were included [30].

### Histopathological analysis

Cardiac tissue samples fixed in 10% formalin-PBS were processed and embedded in paraffin. Four 3 µm sections were prepared for each animal: two were stained with hematoxylin-eosin stain and two with Masson's Trichrome stain. All sections were of approximately equal in size. Inflammatory infiltrate was classified as described previously [25]: Absent; focal or mild myocarditis (lymphocytes seen in 2–15% of the entire section); moderate (20–60%); or severe myocarditis (>70%). Mild myocarditis was focal; moderate and severe inflammation was either multi-focal or diffuse. Degrees of necrosis were classified as follows: absent; mild (necrosis in 5%–15% of cardiomyocytes in the entire section); moderate (16–45%); or severe (>45%). The quantification of parasites was based on the mean number of amastigotes seen in the entirety of the two examined sections: absent (no parasites seen), rare (one amastigote nest), moderate (2–10 nests), or abundant (more than 10 nests).

### Immunohistochemical detection of collagen isotypes

The semiquantification of collagen I, collagen III and collagen IV were performed by IHC. Cardiac tissue samples fixed in formalin-PBS were embedded in paraffin and serial sections of 3 µm were cut. After deparaffinization and hydration steps, the sections were digested with 15 ug/ml proteinase K (Invitrogen, USA) at 37°C for 15 min. Blocking steps were performed using 3% hydrogen peroxide and 5% fetal bovine serum. Rabbit polyclonal antibodies (Cosmo Bio Co, Ltd-USA) against collagens type I, III and IV were used at a dilution of 1/100. An ABC staining kit (Dako, USA) was used for the subsequent procedures. The sections were incubated with diaminobenzidine-hydrogen peroxide kit (Dako, USA) and then counterstained with hematoxylin. The determination of levels of collagen was performed with an optical microscope at 1000×; four quadrants were drawn on cardiac tissue samples and 15 microscopic fields were read in each quadrant. Collagen levels deposition was classified as: normal; mild (focal increase in collagen in 5–25% of fields); moderate (focal increase in 26%–40% of fields); or severe (diffuse increase in >40% of fields).

### Detection of levels of pro-collagen I and pro-collagen III in serum

Levels of carboxy terminal propeptide of type I pro-collagen and N-terminal peptide of type III pro-collagen were measured by ELISA (MyBiosource, USA). Limit of detection was 0.1 ng/ml.

### Detection of apoptosis in situ

Quantification of apoptotic cells was performed by Terminal Deoxynucleotidyl Transferase dUTP nick end labeling (TUNEL) (Roche Laboratories, USA) according to manufacturer's instructions with some modifications. Briefly, sections of 3 µm were obtained from cardiac tissue stored in ethanol and embedded in paraffin. After deparaffinization and hydration steps, sections were permeabilized with 0.1% triton X-100, blocked with 3% H_2_O_2_, and 5% skim milk (Nestle, Peru) and 2% BSA (Sigma, USA). TUNEL reaction was used at a 1/18 dilution. The nucleus were also stained using 5 µg propidium iodide (Biovision, USA) and 300 nM DAPI (Invitrogen, USA). Two adjacent sections of block were used for detection of apoptosis; one section was stained for detection of apoptotic cells using TUNEL as described above and the other was stained for H&E staining to aid in differentiation of the cell types implicated in apoptosis. The sections were examined by fluorescent and optical microscopy at 1000×. Thirty microscopic fields were read in each tissue. The number of apoptotic cells and non-apoptotic cells was counted in each field to determine the percentage of apoptotic cells.

### Statistical methods

The associations between categorical variables were assessed by chi-square test. The differences in mean levels of apoptotic cardiomyocytes, PICP and PIIINP between guinea pigs with cardiac fibrosis and without cardiac fibrosis were examined by T-Test student with unequal variances. All the calculations were made using the software STATA version 10 (Stata Corp., College Station, TX, USA), p values less than 0.05 were considered significant.

## Results

### *T. cruzi* presence in cardiac tissue

Amastigote nests in cardiac tissue were observed in specimens collected from 20 dpi until 365 dpi. Parasites in cardiac tissue were judged as abundant (mean ± SD: 41±23.6, amastigote nests in the two entire sections) at 25 dpi, but mild or moderate at other time points. Parasites were seen between cardiac fibers and were surrounded with lymphocytes, but most inflammatory cells were distant from the parasites.

The percentage of animals with amastigote nests and positive kDNA PCR results varied by phase: acute phase (62.5% with nests, 100% positive by PCR), early chronic phase (87.5%, 75%), chronic phase (62.5%, 75%). In the chronic phase (365 dpi), the presence of kDNA in cardiac tissue (6/8) was significantly associated with cardiac fibrosis (5/8) (p = 0.035).

### Levels of IgG1 and IgG2 against *T. cruzi* antigens

IgG1 anti-*T. cruzi* levels (an indicator of Th2 immune response) were detected from 55 dpi until 365 dpi with a peak at 165 dpi. IgG2 anti- *T. cruzi* levels (an indicator of Th1 immune response) were detected starting at 20 dpi and peaked at 55 dpi. Levels of IgG2 anti-*T. cruzi* were always higher (by 1.5–7.5-fold) than levels of IgG1 anti-*T. cruzi*.

### Necrosis and apoptosis in cardiac tissue

**Figure 1:**
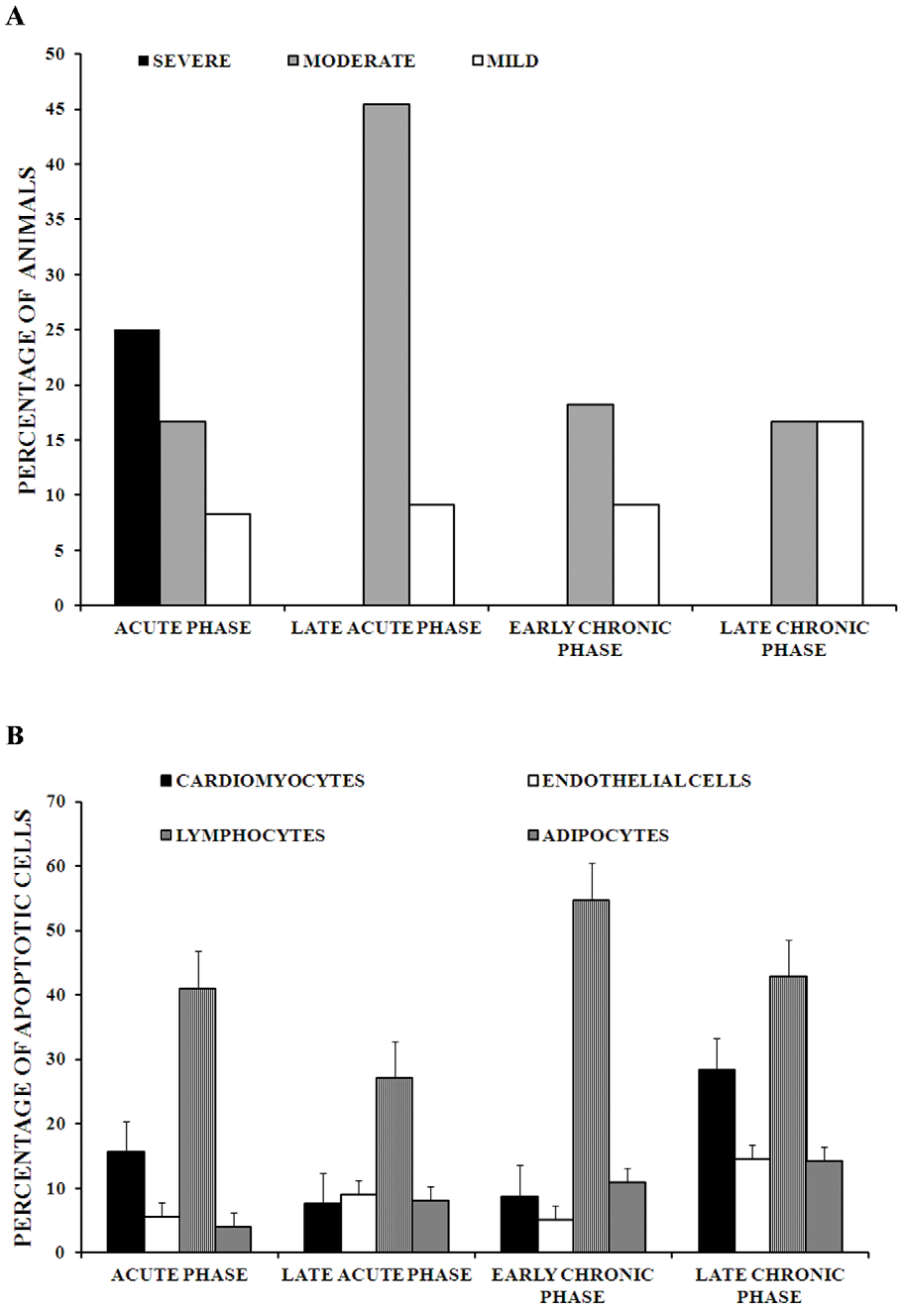
Necrosis and apoptosis during cardiac remodeling in guinea pig infected with *T. cruzi*. 1A. Degrees of necrosis in cardiac tissue of guinea pig infected with *T. cruzi*. Degrees of necrosis were determined semiquantitatively: Mild (5%–15% of cardiomyocytes of the entire section), 3. Moderate (16–45% of cardiomyocytes of the entire section) and 4. Severe (more than 45% of cardiomyocytes of the entire section). 1B. Percentages of number of apoptotic cells in cardiomyocytes, lymphocytes, endothelial cells and adipocytes of cardiac tissue of guinea pig infected with *T. cruzi*. Percentage of cells with apoptosis was determined in 30 microscopy fields. Acute phase: n = 12, late acute phase: n = 11, early chronic phase: n = 11 and chronic phase: n = 6. The bars and error bars represent mean ± standard errors.

Necrosis of cardiomyocytes was observed at each time point over the course of infection in higher degree than apoptosis. Severe (more than 45% of cardiomyocytes) and moderate (16%–45% of cardiomyocytes) necrosis were seen more often in the acute phase; in the chronic phase, most necrosis was moderate (17%) or mild (5%–15% of cardiomyocytes) (Figure 1A).

**Figure 2:**
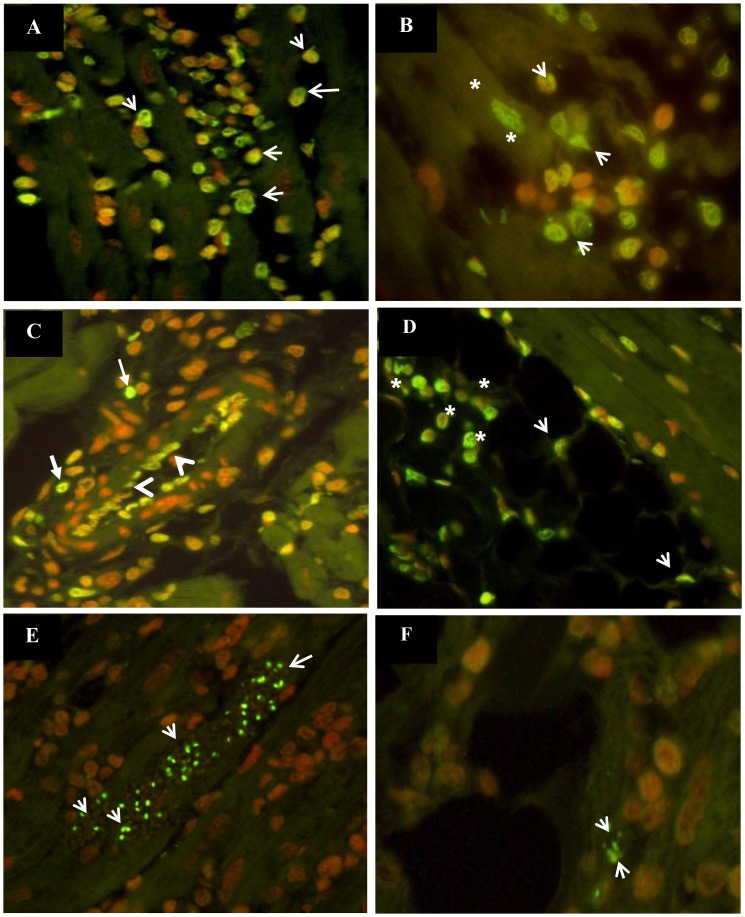
Detection of apoptotic cells in cardiac tissue of guinea pigs infected with *T. cruzi*. (A). Apoptosis in inflammatory infiltrate (arrows) at 165 dpi, 400×. (B) Apoptosis of cardiomyocytes (asterisks) surrounded by inflammatory infiltrate (arrows) at 365 dpi, 1000×. (C). Apoptosis of endothelial cells (arrowheads) and inflammatory infiltrate (arrows) at 25 dpi, 400×. (D). Apoptosis of epicardial adipose tissue (arrowheads) and inflammatory cells (asterisks) at 25 dpi, 400×. (E). Apoptosis-like death (green nucleus) in amastigote nests at 25 dpi, 400×. (F). Apoptosis-like death in amastigote nest (arrowheads) detected in cardiac tissue and surrounded by adipocyte cells and inflammatory infiltrate at 25 dpi, 400×. Apoptotic cells: green or yellow nucleus). Non-apoptotic cells: red nucleus.

Apoptosis was rare in uninfected animals (0–1.9% of cells apoptotic). By contrast, substantial levels of apoptosis were observed in cardiomyocytes (8%–28.4% cells apoptotic), endothelial cells (5.2%–14.6%), epicardial adipose cells (3.3%–14.3%) and lymphocytes (27.2%–54.7%) in cardiac tissue throughout the course of *T. cruzi* infection (Figure 1B). The highest percentage of apoptotic cells were seen among lymphocytes (Figure 1B and 2a). Apoptosis of cardiomyocytes, endothelial cells and epicardial adipocytes was most frequent during the chronic phase (28.4%, 14.6% and 14.3% of cells apoptotic, respectively). Generally, apoptotic cardiomyocytes, endothelial cells and epicardial adipocytes were observed near inflammatory infiltrate, but distant from amastigote nests (Figure 2b, 2c and 2d). Apoptosis-like death was observed in all amastigote nests, both large and small, throughout the acute and chronic phases of infection (Figure 2e and 2f).

### Histopathological changes in epicardial adipose tissue

**Figure 3:**
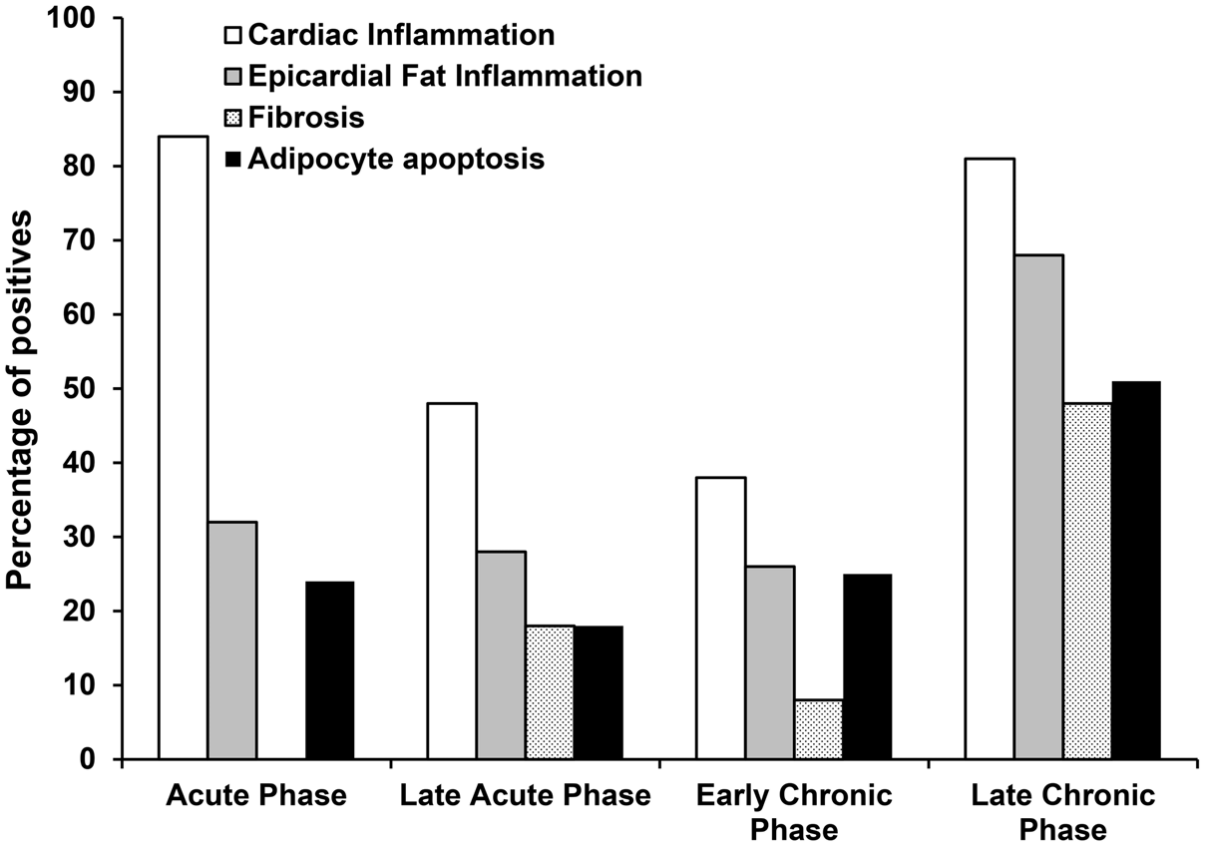
Histopathological changes in cardiac tissue of guinea pigs infected with *T. cruzi*. Percentage of *T. cruzi* infected guinea pigs with presence of cardiac inflammation, epicardial fat inflammation, fibrosis and adipocyte apoptosis. Acute phase: n = 12, late acute phase: n = 11, early chronic phase: n = 11 and chronic phase: n = 6.

During the chronic phase, areas of adipose tissue between cardiac fibers, often with neighboring inflammatory cells were seen in tissue from 33% (2/6) of infected guinea pigs. Areas of mild to moderate inflammation were observed in epicardial adipose tissue (EAT) and in the cardiac tissue adjacent to it in 55% (22/40) of infected guinea pigs throughout the course of the infection, with highest frequency in the chronic phase (66.7%) (Figure 3), these areas of inflammation were always surrounded by large areas of fibrosis. No parasites were observed in epicardial adipose tissue of guinea pigs.

### Detection of collagen isotypes in cardiac tissue

**Figure 4:**
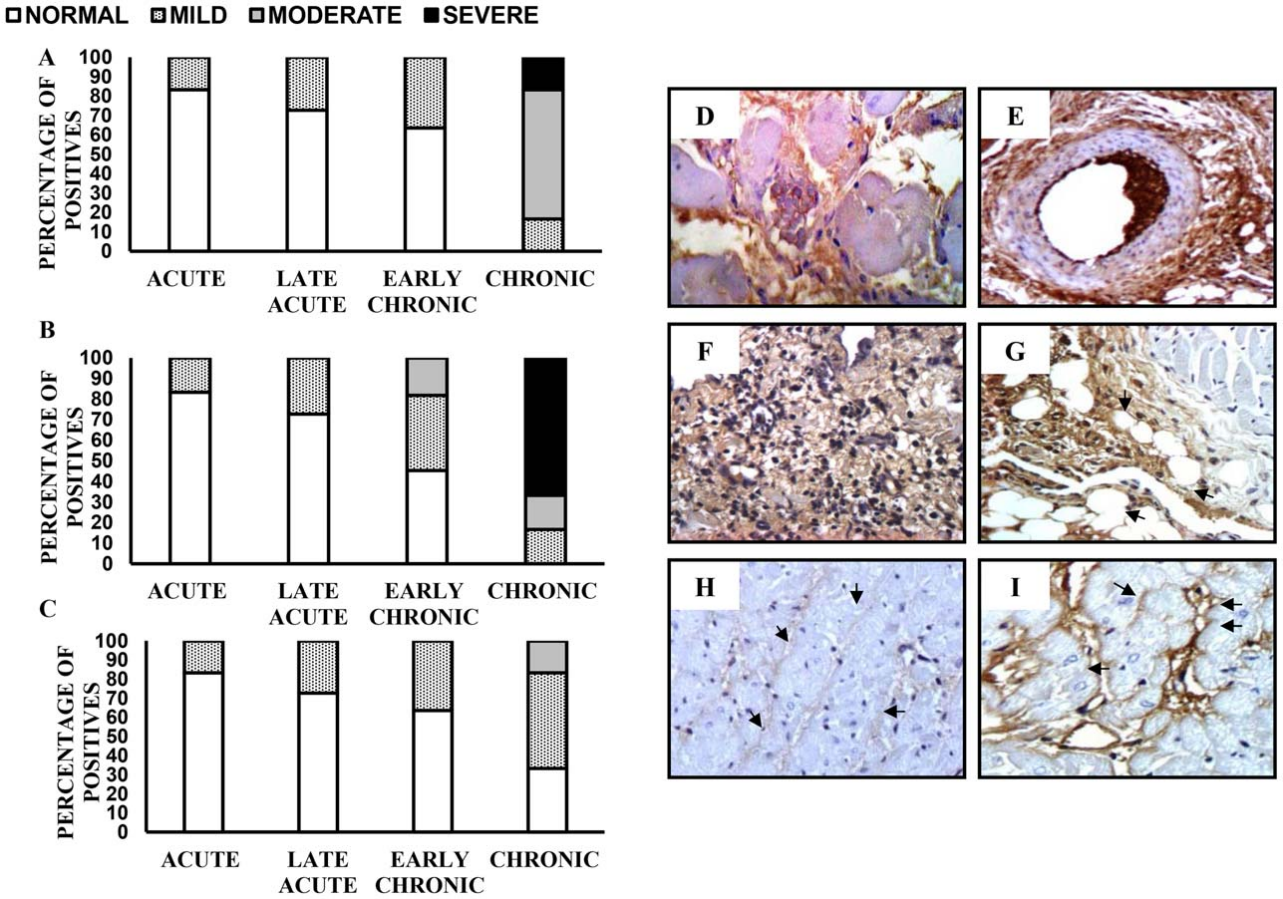
Distribution of collagen isotypes in cardiac tissue of guinea pig infected with *T. cruzi*. Deposition of collagen I (A), collagen III (B) and collagen IV (C) during the course of infection. Moderate increase of interstitial collagen I, at 365 dpi, 400× (D). Increase of perivascular collagen I, at 365 dpi, 400× (E). Severe increase of interstitial collagen III and moderate inflammation, at 365 dpi, 400× (F). Increase of collagen III near epicardial adipose tissue at 165 dpi, 400× (G). Detection of collagen IV in non-infected guinea pig (H). Increase of collagen IV in the basement of cardiac fibers, at 365 dpi, 400× (I). Acute phase (20–25 dpi): n = 12, late acute phase (40–55 dpi): n = 11, early chronic phase (115–165 dpi): n = 11 and chronic phase (365 dpi): n = 6.

Isotypes of CI, CIII and CIV were detected in cardiac tissue by immunohistochemistry. From the acute to the early chronic phase (115–165 days pi), there was a mild to moderate increase in the levels of all three types of collagen. A moderate to severe increase in levels of CI, CIII and CIV was observed during the chronic phase (365 days pi), with CIII being present in highest levels (Figure 4a, 4b and 4c). CI and CIII were detected in interstitial and perivascular spaces (Figure 4d, 4e and 4f). The deposits of collagen were near inflammatory cells such as lymphocytes (Figure 4f). Deposits of CIII were also observed close to EAT, usually surrounded by inflammatory cells (Figure 4g). CIV was detected in some interstitial forms and on the basement membrane of cardiomyocytes (Figure 4h and 4i).

### Serum levels of PICP and PIIINP and their relationship with fibrosis in cardiac tissue

**Figure 5:**
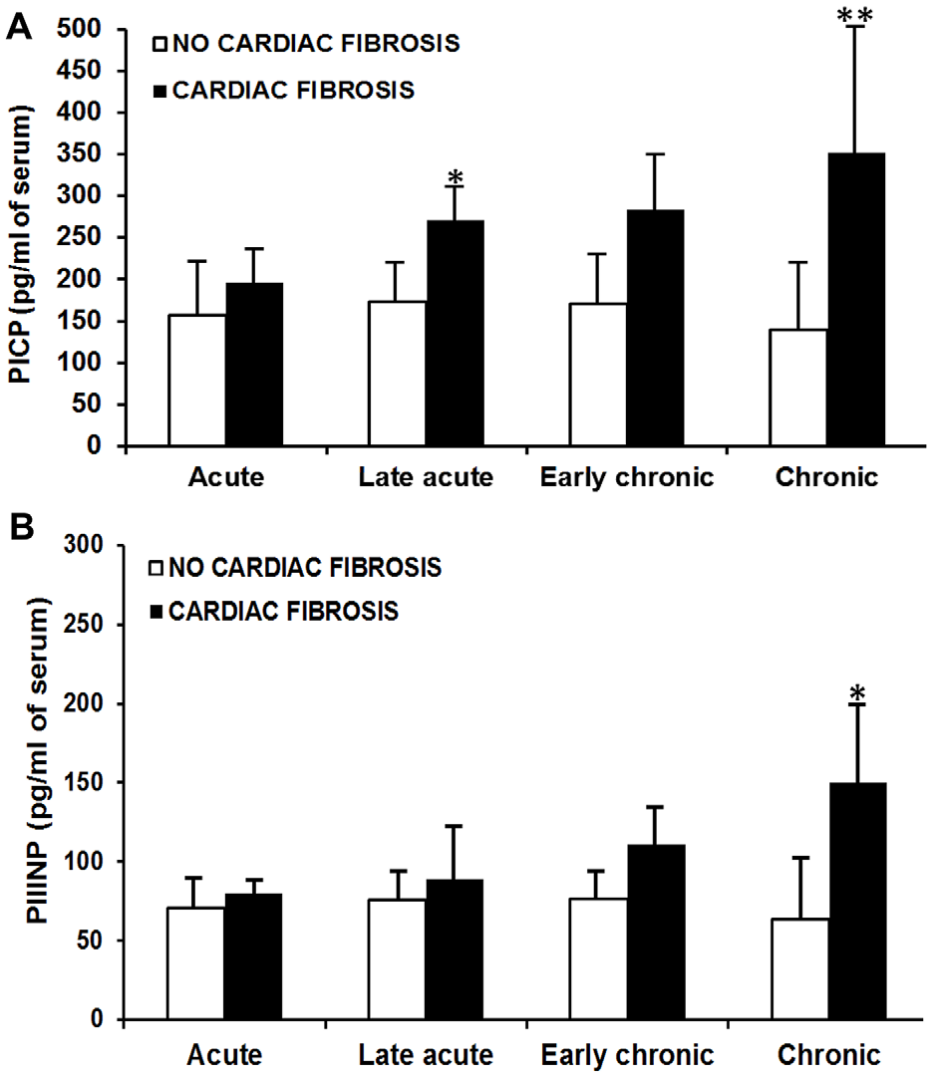
Serum levels of PICP and PIIINP in guinea pig infected with *T. cruzi*. A. Serum levels of procollagen type I carboxy-terminal propeptide (PICP) were significantly higher in guinea pigs with cardiac fibrosis than those without fibrosis: * p = 0.019. ** p = 0.022. Fibrotic cases were considered those with more than 15% of microscopic fields with collagen I deposition. B. Serum levels of procollagen type III amino-terminal propeptide (PIIINP) significantly higher in guinea pigs with cardiac fibrosis than those without fibrosis: * p = 0.028. Fibrotic cases were considered those with more than 15% of microscopic fields with collagen III deposition. Acute phase: n = 12, late acute phase: n = 11, early chronic phase: n = 11 and chronic phase: n = 6. The bars and error bars represent mean ± standard deviation. T-test with unequal variances.

Serum levels of PICP and PIIINP were slightly increased during the acute phase. Levels of both PICP and PIIINP were increased in infected animals compared to non-infected animals during the chronic phase. High levels of PICP and PIIINP were associated with the presence of cardiac fibrosis during the course of the infection (p<0.05) (Figure 5).

## Discussion

Knowledge of the mechanisms involved in *T. cruzi*-induced cardiac pathology is an essential step toward improved understanding of pathogenesis at the cellular level and identification of useful biomarkers of cardiac progression. Serum biomarkers would provide valuable new tools for prognosis and to evaluate new drug candidates aimed at halting progression of CCHD. In the present study, the guinea pig model of *T. cruzi* infection reflected the known pathology of Chagas cardiomyopathy, including parasite persistence associated with cardiac fibrosis, apoptotic cell death, activation of Th1/Th2 immune response, and increased collagen I, III and IV in the chronic phase. Serum levels of PICP and PIIINP correlated well with fibrosis and could serve as potential biomarkers for cardiac disease progression in patients. Furthermore, our data suggest that epicardial fat may play a role in the pathology of CCHD.

The high levels of anti-*T.cruzi* IgG2 in the earliest stages of infection suggest that the guinea pig activates an early Th1 response [31] that succeeds in limiting parasite replication, leading to the transition to the chronic phase. These findings demonstrate a process similar to that observed in the chronic Chagas disease model using C57BL/6 mice, a mouse strain that activates a Th1 response and exercises efficient control of the acute infection [32]. However, in the chronic phase of the guinea pig model, the Th2 response (IgG1) was also high. This response may contribute to cardiac fibrosis; the Th2-type antibody response in BALB/c mice is associated with intraventricular conduction abnormalities, sinus bradycardia and widening of the interfibrillar space [33].

The results of this study showed that although necrosis is the main mechanism of cardiomyocyte death throughout *T. cruzi* infection, apoptosis is a significant contributor to cardiomyocyte loss in the chronic phase. Apoptosis was observed in cardiomyocytes, endothelial cells and adipocytes close to inflammatory cells but remote from parasites, suggesting that release of pro-inflammatory cytokines such as IL1β, INFγ and TNFα may be involved in triggering apoptosis in this setting. The role of cardiomyocyte death by apoptosis in CCHD is still uncertain, but apoptosis has been demonstrated in other dilated cardiomyopathies [34]–[35]. Diverse mechanisms have been described, including activation of protein kinase B signaling, cytochrome c and β-adrenergic receptor pathways, and increased Fas receptor signaling by up-regulation of TNF-α and degradation of inhibitors of Fas receptor signaling [36]. However, because TUNEL staining is not limited to the detection of apoptotic cells [37], further studies are needed to define the impact of apoptosis in cell loss during CCHD.

Apoptosis-like death has been reported in *T. cruzi* amastigote nests and trypomastigotes, and hypothesized to be the result of control of parasite burden regulated by the parasite itself or by the host, parasite evasion of the host immune response and clonal selection [13],[38]–[39]. However, these studies were conducted during the acute phase [13],[38], whereas we observed apoptosis-like death in nests during both the acute and chronic phases. We have observed apoptosis in small and large amastigote nests, providing evidence against the hypothesis that contact between amastigotes triggers apoptosis as a mechanism to limit intracellular population size [40]. This finding is more consistent with apoptosis in *T. cruzi* being viewed as a developmentally regulated process, with the activation of different apoptotic mechanisms to allow altruistic survival of the parasite population [39]. The fact that non-apoptotic cells were observed near the parasites even in the presence of inflammatory cells suggests that molecules released by the parasite such transialidases and/or cruzipain could be involved in inhibition of apoptosis in the host cell [41]–[43]. Apoptosis of parasites has been proposed as an important *T. cruzi* virulence factor, in which stimulation by apoptotic amastigotes leads to anti-inflammatory cytokine production favoring parasite persistence [44].

Although we did not observe parasites in epicardial adipose tissue (EAT), our results demonstrate that inflammation and apoptosis of EAT is an important characteristic of *T. cruzi* infection in the guinea pig model. EAT is thought to affect cardiac function through paracrine and endocrine regulation [45]. In metabolic and cardiovascular disease states, EAT reduces the production of cardioprotective adipocytokines, such as adiponectin, and induces the production of detrimental pro-inflammatory adipocytokines such as leptin, resistin, IL-6, tumor necrosis factor-α, and IL-17. The resulting inflammatory state alters the balance between vascular nitric oxide, endothelin-1, and superoxide production, promoting vasoconstriction [46],[47]. However, the role of EAT in the pathogenesis of CCHD is still unknown, in part because the more widely used animal models, such as mice and rats, have little or no EAT [46]. The presence of *T. cruzi* has been demonstrated in subcutaneous adipose tissue in chronic chagasic patients [48] and in brown and white adipose tissue of mouse [49],[50]. The parasite is also known to induce an inflammatory response in adipocyte culture [51], and in the murine model, infection leads to increased levels of inflammatory adipocytokines and decreased levels of anti-inflammatory adipocytokines [49],[50]. The presence of this tissue and the demonstration of *T. cruzi*-induced pathology make the guinea pig an attractive alternative model to study this feature of Chagas disease.

We also found replacement of cardiac fibers by adipose tissue close to areas of collagen accumulation, similar to the cardiac histopathology observed in dogs with chronic *T. cruzi* infection [52]. This myocardial adipose tissue may contribute to hyperactivation of β-oxidation of fatty acids leading to excess formation of reactive oxygen species and modulation of the sarco/endoplasmic reticulum Ca2+-ATPase, an early contributor to diastolic dysfunction in myocardial fibrosis and hypertrophy [53].

We observed an increased collagen deposition (CI, CIII and CIV) in cardiac tissue throughout the course of the infection, with CI and CIII in interstitial and perivascular spaces, and CIV in interstitial spaces and on the basement membrane of cardiomyocytes. These results resemble those of the murine model of chronic Chagas disease [19]. We performed all examinations with blinded observers and in duplicate to minimize observer bias. Nevertheless, the use of automated image analysis may provide more accurate quantification of collagen isotypes.

Serum levels of PICP are used as an indicator of collagen I synthesis, based on the release of one molecule of PICP during the synthesis of each molecule of collagen I [54]. Serum levels of PICP have been used as an indicator of the severity of cardiac fibrosis in hypertensive patients [55]. In our data, collagen deposition in heart tissue was significantly correlated with elevated levels of PICP from the late acute phase through the late chronic phase. In the early acute phase, mild levels of collagen I deposition (5–15% of microscopic fields with collagen deposition), but serum PICP levels were not significantly elevated. Some authors have observed that PICP may reflect the rate rather than the quantity of collagen I deposition [54],5[6]. Other potential explanations include suboptimal sensitivity of the serum PICP ELISA [54],[55] or the prolonged time over which the collagen is synthesized; because it has been demonstrated that large quantities of collagen may be deposited in the absence of elevated circulating markers of collagen synthesis [56]. PICP rose significantly in the late acute phase, suggesting an increase in collagen I synthesis following the severe inflammation and necrotic cell death in the early acute phase [21],[57]. Subsequently, activation of anti-inflammatory mechanisms (indicated by high levels of IgG1), induction of cell death by apoptosis and necrosis, and the persistence of the parasite may lead to activation of growth factors such as TGFβ1 which stimulate collagen synthesis resulting in intensified deposition of collagen in the chronic phase [56],[57]

PIIINP is reported to be a useful marker of collagen III synthesis in patients with dilated cardiomyopathy [58], hypertrophic cardiomyopathy [59] and heart failure [60]. In one study, high circulating PIIINP was shown to predict mortality in patients with dilated cardiomyopathy [53]. In contrast to PICP, the relationship between the number of molecules of PIIINP released to the circulation and the number of collagen III molecules synthesized is thought to be variable [54]. PIIINP is not completed eliminated from procollagen during the synthesis of collagen III and the remaining PIIINP may be eliminated from the collagen fiber during degradation. Thus serum levels of PIIINP reflect both collagen III synthesis and degradation [54]. In the late chronic phase we observed a significant increase in serum PIIINP together with severe collagen III deposition by microscopy, suggesting that in this phase collagen III synthesis predominates over degradation.

In summary, our data indicate that PICP and PIIINP are promising biomarkers of cardiac collagen metabolism during the development of Chagas heart disease, and that there may be a contribution to cardiac cell death through apoptosis as well as necrosis. Future studies designed to relate the levels of PICP and PIIINP with cardiac function are needed to determine its potential value for CCHD prognosis and measurement of response to therapy. Further studies could be useful to evaluate whether inhibition of apoptosis could slow the development of cardiomyopathy in patients with chronic Chagas disease.
